# Heat stroke admissions during heat waves in 1,916 US counties for the period from 1999 to 2010 and their effect modifiers

**DOI:** 10.1186/s12940-016-0167-3

**Published:** 2016-08-08

**Authors:** Yan Wang, Jennifer F. Bobb, Bianca Papi, Yun Wang, Anna Kosheleva, Qian Di, Joel D. Schwartz, Francesca Dominici

**Affiliations:** 1 Department of Environmental Health, Harvard T.H. Chan School of Public Health, 401 Park Drive, Boston, MA 02215 USA; 2 Biostatistics Unit, Group Health Research Institute, 1730 Minor Ave #1600, Seattle, WA 98101 USA; 3 Department of Biostatistics, Harvard T.H. Chan School of Public Health, 677 Huntington Ave, Boston, MA 02115 USA

**Keywords:** Heat wave, Heat stroke, Medicare beneficiaries, Spatiotemporal variation, Effect modification

## Abstract

**Background:**

Heat stroke is a serious heat-related illness, especially among older adults. However, little is known regarding the spatiotemporal variation of heat stroke admissions during heat waves and what factors modify the adverse effects.

**Methods:**

We conducted a large-scale national study among 23.5 million Medicare fee-for-service beneficiaries per year residing in 1,916 US counties during 1999–2010. Heat wave days, defined as a period of at least two consecutive days with temperatures exceeding the 97th percentile of that county’s temperatures, were matched to non-heat wave days by county and week. We fitted random-effects Poisson regression models to estimate the relative risk (RR) of heat stroke admissions on a heat wave day as compared to a matched non-heat wave day. A variety of effect modifiers were tested including individual-level covariates, community-level covariates, meteorological conditions, and the intensity and duration of the heat wave event.

**Results:**

The RR declined substantially from 71.0 (21.3–236.2) in 1999 to 3.5 (1.9–6.5) in 2010, and was highest in the northeast and lowest in the west north central regions of the US. We found a lower RR among counties with higher central air conditioning (AC) prevalence. More severe and longer-lasting heat waves had higher RRs.

**Conclusions:**

Heat stroke hospitalizations associated with heat waves declined dramatically over time, indicating increased resilience to extreme heat among older adults. Considerable risks, however, still remain through 2010, which could be addressed through public health interventions at a regional scale to further increase central AC and monitoring heat waves.

**Electronic supplementary material:**

The online version of this article (doi:10.1186/s12940-016-0167-3) contains supplementary material, which is available to authorized users.

## Background

A heat wave, characterized by a sustained period of extreme hot weather, is associated with increased mortality and morbidity, particularly among older adults [1–5]. During 2000–2009, heat waves contributed to over five billion dollars of health cost in the US [6]. Under the changing climate, the intensity, frequency, and duration of heat waves have increased especially in the past 15 years [7, 8] and are very likely to continue to increase in this century [9, 10]. A recent national survey, however, found that response plans for extreme hot temperature were far from adequate in the US [11]. A more in-depth understanding of the spatiotemporal pattern of heat-related diseases and the factors that can attenuate the adverse health impact of heat waves is essential for the development of regional public health policies to protect susceptible population from the adverse effect of heat waves.

Heat stroke is one of the most serious and life-threatening illnesses directly related to heat exposure [12]. Heat stroke patients typically have an increased core body temperature of over 40 °C and dysfunction of the central nervous system, which is often fatal if the treatment is not adequate [13]. Older adults are particularly susceptible due to decreased thermoregulatory function, chronic health condition, or medication use that interferes with the functioning of thermoregulation [14]. Although several studies have investigated the association between heat exposure and heat strokes [2, 3, 15–17], most of these were restricted to a small geographical region. One national study, Bobb et al. (2014), estimated the relative risk (RR) of a composite outcome of heat-related diseases on heat wave days compared to non-heat wave days but only reported a national overall estimate for the study period 1999–2010 [2]. Such an overall estimate, however, masks potentially substantial spatial and temporal variability in the risk. To our knowledge, no national study has examined whether heat stroke admissions during heat waves vary across geographical region or over time. Importantly, little is known about what factors explain spatial and temporal heterogeneity in heat stroke risk.

We estimated the RR of heat stroke hospital admissions associated with heat waves using a large nationwide database of unprecedented size and accuracy. We hypothesized that the RR might be changing over time, that it would vary across geographical regions, and that several individual- and county-level variables, meteorological conditions, as well as the characteristics of the heat wave (i.e., intensity and duration) would explain the spatial and temporal variability in the heat wave-related risks.

## Methods

### Study population

Our study population comprised Medicare beneficiaries who were aged 65 years or older and enrolled in the fee-for-service program for at least one month from January 1st, 1999 to December 31st, 2010 in the contiguous US [2]. In total, 23.5 million participants per year residing in 1,916 counties were included. For each of the eligible Medicare beneficiaries, data on age, county of residence, dates of hospital admission, and the International Classification of Diseases, Ninth Revision (ICD-9) code for the primary cause of each hospitalization were extracted.

The outcome was hospital admissions with a principal discharge code of the ICD-9, 992.0 denoting heat stroke and sunstroke (we will refer to both of these as heat stroke). For each county, we calculate the daily number of hospital admissions for heat stroke in (numerators) and the daily number of Medicare beneficiaries (denominators).

### Meteorological data and heat wave definition

Monitoring data for daily mean temperatures were obtained from National Climatic Data Center (NCDC, now National Centers for Environmental Information (NCEI); Global Summary of the Day). Daily temperature in a county was averaged from daily temperatures of all monitoring sites within that county. For counties (on days) without monitoring sites or temperature data available, daily temperature was obtained by averaging over monitoring sites within 35 km of the county’s centroid [18]. Counties were excluded from the analysis if they (1) did not have at least one temperature monitoring site within the county and (2) did not have a monitor within 35 km from the county’s centroid. The same method was applied to obtain the average daily dew point temperature, relative humidity (RH), and wind speed for each of the counties.

The primary definition of a heat wave event was a period of at least two consecutive days with daily mean temperature greater than the 97th percentile of temperatures in that county. We did not restrict the dataset only to the summer season. We compared the estimates obtained based on this definition to those obtained using stricter definitions, which included >98th, 99th for two consecutive days and >97th, 98th, 99th for four consecutive days [2]. We chose >97th for two consecutive days as the main exposure is because such a definition is able to capture more heat stroke hospital admissions than other stricter definitions, and therefore increase the precision of the heat wave risk estimates.

### Covariates

Monthly Moderate Resolution Imaging Spectroradiometer (MODIS) Level 3 Normalized Difference Vegetation Index (NDVI) data on a 1 km by 1 km grid were obtained from National Aeronautics and Space Administration (NASA) from February 2000 to December 2010. The NDVI product is a measure of the green leaf vegetation (greenness) of the grid cell, with values from lowest to highest vegetation ranging from −0.2 to 1.

Daily levels of ozone were obtained from Environmental Protection Agency (EPA) and the Interagency Monitoring of Protected Visual Environment (IMPROVE) monitoring sites during 1999–2010.

Daily cloud cover data (low, medium, and high cloud cover) were obtained from National Center for Environmental Protection (NCEP) – National Center for Atmospheric Research (NCAR) North American Regional Reanalysis data set, which have a spatial resolution of approximately 0.3°.

AC prevalence in metropolitan areas was obtained from American Housing Survey (AHS) data from 1998 to 2011 (http://www.census.gov/programs-surveys/ahs/data.html). We considered both prevalence of central AC and of “any AC”, defined as having either central AC or one or more room units.

More details about these covariates are described in Additional file 1: Text 1.

### Statistical analysis

**Fig. 1:**
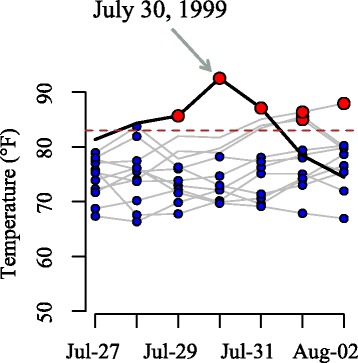
An example illustrating how control days were matched to heat wave days. July 30, 1999 was a heat wave day in Chicago. Five candidate control days occurred in the same county and same week of the year as this heat wave day. One of them was randomly selected and matched with the heat wave day

Each heat wave day was matched to a non-heat wave (control) day by county and by week (Fig. 1). Specifically, for each heat wave day, a candidate non-heat wave day was a day that was: 1) in other years as the index heat wave day; 2) in the same week and county as the index heat wave day; and 3) not within three days of a different heat wave day. If there are multiple candidate control days, we select one of them at random to achieve 1:1 matching [2]. Matching is a well-established approach to achieve balance in the covariates distribution between heat wave days and non-heat wave days. By design, the matching eliminated potential confounding by seasonality and county-level time-invariant covariates since the matching results in a similar distribution of weeks during the study period and county-level covariates in the matched non-heat wave days as the distribution of those factors among the heat wave days. Matching also substantially reduced the size of the data, thereby increasing computational efficiency.

Using the matched dataset, we fitted a random-effects Poisson regression model with county-level random intercepts, controlling for calendar year and day of the week. More formally, in county c on day t, we assumed that the number of hospital admissions for heat stroke (Y_ct_) follows

1 $$ \log\ \mathrm{E}\left({\mathrm{Y}}_{\mathrm{ct}}\right)={\gamma}_{0,\mathrm{c}}+{\beta}_0+{\beta}_1{\mathrm{HW}}_{\mathrm{ct}}+{\beta}_2{\mathrm{Dow}}_{\mathrm{t}}+{\beta}_3{\mathrm{Y}\mathrm{ear}}_{\mathrm{t}}+ \log \left({\mathrm{P}}_{\mathrm{ct}}\right) $$

where γ_0,c_ is the random intercept for county c, HW_ct_ indicates if day t in county c is a heat wave day, Dow_t_ is an indicator variable for day of the week, Year_t_ is a categorical variable for year, and P_ct_ is the number of Medicare enrollees in county c on day t. We are interested in the adjusted relative risk (RR) of heat stroke admissions on heat wave days compared to matched non-heat wave days, exp (β_1_).

We estimated a long-term time trend of the RR during 1999–2010 in two different ways. First, we included interaction terms between the heat wave and the indicator variables for each year. Second, we smoothed the trend using interaction terms between the heat wave day indicator and natural cubic spline of year with five degrees of freedom.

To test whether the RR differed by month of the summer season, we included interaction terms of the heat wave day indicator with indicator variables for month (June, July, and August).

We assigned each of the 1,916 US counties to one of the nine climate regions [19]: central, east north central, west north central, northeast, northwest, south, southeast, southwest, and west. We estimated the RR for each climate region by a product term between heat wave and a categorical variable for climate regions.

A variety of effect modifiers were tested. We used age as an indicator of individual-level vulnerability and tested if it modifies the association between heat waves and heat stroke. A number of county-level variables were also tested, including AC prevalence (central or any), mean summer NDVI, mean summer ozone concentration, mean summer temperature, mean summer RH, mean summer wind speed, mean summer low/medium/high cloud cover, and urbanicity. In addition, we also tested if (1) the intensity and the duration of heat waves, (2) the meteorological conditions on heat wave days (temperature, dew point, RH, cloud cover), and (3) the temperature percentile on the day before a heat wave day altered the association. More details about the modeling approach are described in Additional file 1: Text 2.

Several sensitivity analyses were conducted. First, we additionally adjusted for a natural spline of day of the year with six degree of freedom in the main model. Second, we eliminated the observation if the mean temperature of that day was computed using less than 18 h of measurement data. Third, we eliminated the counties whose temperatures were assigned using the 35 km rule (38 %). Fourth, we estimated the regional RRs using two other definitions temperatures >98th and >99th percentile for at least two consecutive days and >97th for at least four consecutive days. Fifth, we fitted quasi-Poisson models to estimate both the overall and the regional RRs.

## Results

There were 119,817 heat wave days in 1,916 counties during 1999–2010. Table 1 presents the number of heat stroke admissions on heat wave days and matched non-heat wave days by age group. The overall RR of heat stroke on heat wave days (>97th, two days) compared to matched non-heat wave days was 11.0 (95 % confidence interval: 8.8–13.6).

**Table 1:** Number of heat stroke hospital admissions on heat wave days (primary definition) and matched non-heat wave days by age group

Age group	Heat wave days	Matched non-heat wave days
65–74	331	27
75–84	450	39
>84	349	26

**Fig. 2:**
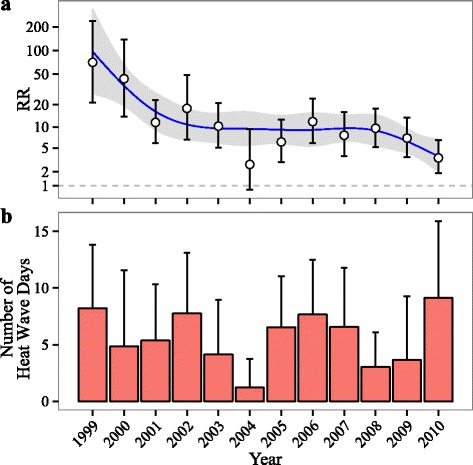
Temporal trend of (**a**) the relative risk (RR) of heat stroke on heat wave days compared to matched control days and (**b**) the average number of heat wave days per county per year over 1999–2010 (the error bars represent one standard deviation). The smoothed trends for the RR were estimated by natural splines with five degrees of freedom. The models controlled for indicator variables for day of the week. The RR (e.g. RR = x) should be interpreted as the risk of heat stroke admissions on heat wave days was x times the risk on matched non-heat wave days

The RR of heat stroke on heat wave days compared to matched non-heat wave days decreased from 71.0 (21.3–236.2) in 1999 to 3.5 (1.9–6.5) in 2010 (Fig. 2). This decrease was the largest in 1999–2003, leveled off in 2003–2008, and then continued to decrease after 2008. Within the summer season, we found that the RR was similar in June and July but was lower in August [p for interaction (August vs. June) = 0.03; p for interaction (July vs. June) = 0.78] (Additional file 1: Figure S1).

**Fig. 3:**
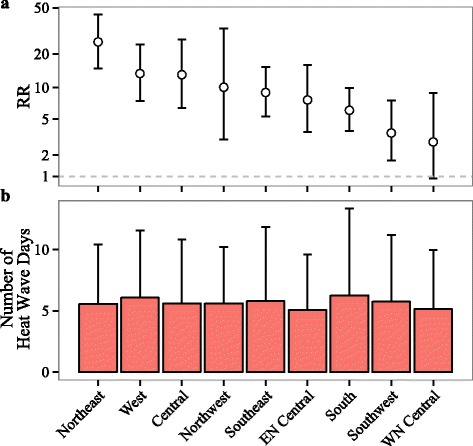
Spatial variation of (**a**) the relative risk (RR) of heat stroke on heat wave days compared to matched control days, and (**b**) the average number of heat wave days per county per year across the climate regions (the error bars represent one standard deviation). The model controlled for indicator variables of year and day of the week

The RR was highest in the northeast [25.5 (14.9–43.6)], followed by the west [13.4 (7.5–24.2)], and lowest in the west north central [2.9 (0.9–8.9)] (Fig. 3). Moreover, the temporal trend of RR during 1999–2010 differed substantially in each of the climate regions (Additional file 1: Figure S2).

The RR was similar across the three age groups [p for interaction (75–85 vs. 65–75) = 0.82; p for interaction (>75 vs. 65–75) = 0.76] (Additional file 1: Figure S3).

**Fig. 4:**
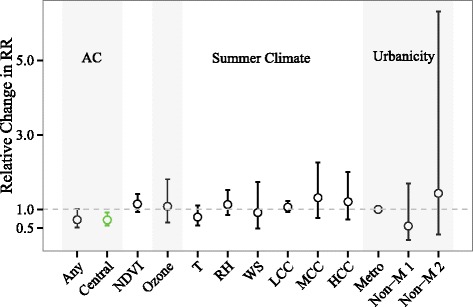
Relative change in the relative risk (RR) of heat stroke on heat wave days compared to matched non-heat wave days associated with changes in county-level covariates: AC prevalence (any and central AC, per 10 % increase), mean summer NDVI (per 0.1 increase), mean ozone concentration (per 10 ppb increase), summer climate [temperature (T): per 10 °F; RH: per 10 %; wind speed (WS): per 0.5 km h^−1^ increase; low cloud cover (LCC), medium cloud cover (MCC), high cloud cover (HCC): per 5 %], and urbanicity [metropolitan (Metro); non-metropolitan with population >20,000 (Non-M 1); non-metropolitan with population < 20,000 (Non-M 2)]. Estimates and error bars in green indicate modifiers that decrease the RR and whose confidence intervals exclude one. The models include both random slopes and random intercepts. The y-axis represents the ratio of the RR per the specified increase in each modifier, obtained as the exponent of the coefficient for the interaction term between the heat wave day indicator variable and the covariate

Counties with higher prevalences of central or any AC had lower heat wave-related RRs. In particular, the RR decreased by 28 % (9 %–43 %) per 10 % increase in central AC prevalence (Fig. 4). NDVI, urbanicity, mean summer temperature, RH, wind speed, cloud cover, or ozone concentration did not modify the association between heat stroke admissions and heat wave days.

**Fig. 5:**
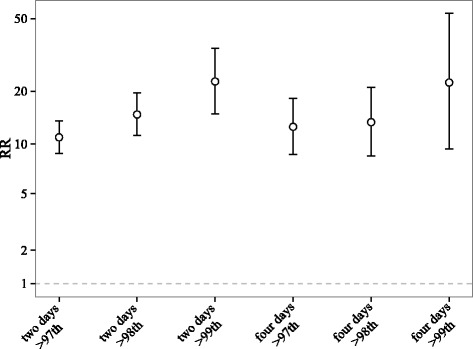
The relative risk (RR) of heat stroke on heat wave days as compared with matched control days by definition of a heat wave event. The definitions of heat wave are temperature >97th, 98th, or 99th percentile for at least two or four consecutive days

The RRs increased with the intensity and duration of heat waves (Fig. 5). Additional file 1: Figure S4 shows that the confidence intervals for the effect modifications of RR by daily meteorological variables all included one. Low temperature (<80th percentile) on the day before heat wave was associated with a higher RR, compared to moderate (p for interaction = 0.05) or high (p for interaction = 0.13) temperature on the day before a heat wave day.

When we further adjusted for a natural cubic spline for day of the year (1-365/366) with six degrees of freedom to account for potential residual confounding by seasonality after matching, the RR did not change [RR 11.0 (8.8–13.6)]. When we eliminated observations with daily mean temperature computed using less than 18 h of the data, we obtained a similar RR of 10.4 (8.3–13.0). When we eliminated counties whose temperature relied on monitoring sites within 35 km but outside of that county, we obtained a similar estimate of 10.3 (8.2–13.0). Additional file 1: Figure S5 presented the regional RR for two stricter definitions >98th and 99th percentile for at least two consecutive days. The relative ranking of RR across regions did not change, whereas the RR increased as the definitions became stricter. We obtained a RR of 11.0 (8.9–13.4) when using a quasi-Poisson model, suggesting the appropriateness of using a Poisson model. The regional RRs using a quasi-Poisson model are also presented in Additional file 1: Figure S6. The confidence intervals are close to the ones presented in Fig. 3a.

## Discussion

We conducted a large-scale national study investigating heat stroke admissions associated with heat waves, including a study population of 23.5 million older adults per year for 12 years and a spatial coverage of 1,916 US counties. We also tested a variety of effect modifications. By understanding which factors explain which locations or subpopulations are most susceptible to the harmful effects of heat can enable targeting public health interventions to those who are most susceptible. This is especially important with the increasing risks of extreme heat events under climate change.

Our study provides evidence on how susceptibility to heat changes both over the course of the warm season, as well as over longer time scales. First, our finding that heat stroke risks declined over time suggests that older adults have become more resilient to the effect of extreme heat on heat stroke over time. This is consistent with previous studies that found declines in heat-related mortality [18, 20, 21]. Although 2002 and 2010 had more heat wave days, the RR continued to decrease suggesting the changing risks were unlikely due to simply lower exposures. The RR was higher in June than in August. This is consistent with some previous studies on mortality suggesting that the health effect of high temperature decreased as summer progressed [22–24]. Future studies could examine whether variables such as the change in dual eligibility over time, change in age structure over time, access to healthcare could explain the temporal trend.

We found that heat stroke risks differed substantially across climate regions and explored whether several county-level covariates might explain this spatial variability. Previous studies provided mixed evidence on whether heat-related morbidity or mortality was modified by AC prevalence [16, 18, 21, 25]. We found strong evidence that counties with higher AC prevalence had greatly reduced risk of heat stroke admissions during heat waves. Although prior work has suggested that vegetation can alter the microclimate and exert a cooling effect by shading the area [26], we did not find a reduction in heat stroke risks in counties with higher NDVI. We found that counties with a warmer summer climate tended to have fewer heat stroke admission associated with heat waves, albeit with wider confidence interval. Counties with warmer climates (e.g. the south) likely have a range of adaptations (e.g., higher AC prevalence in homes or public spaces), and hence may be better prepared for the heat waves. Although urban areas typically have higher temperatures than suburban or rural areas due to the urban heat island effect [27], and although previous work found higher urbanicity to be associated with higher heat-related mortality risk [28], we did not find a clear difference in heat stroke risks by urbanicity (i.e. in metropolitan areas versus non-metropolitan areas). A previous study suggested that ozone pollution positively modifies the relative risk of heat waves on cardiovascular mortality [29]. However, we did not observe interaction between county-average ozone pollution and heat wave.

We found that increased risk of heat stroke admissions during heat waves was larger for more intense or longer-lasting heat waves, consistent with a previous study of heat-related hospitalizations [2] and prior literature on heat-related mortality [28, 30, 31]. In our study, days with higher temperature, dew point temperature, RH, and medium and high cloud cover also tended to have higher risks of heat stroke hospitalizations during heat waves, although the confidence intervals of the effect modification estimates all included zero.

Some evidence has suggested the thermoregulatory system becomes more susceptible to environmental heat exposure as the body ages [13] and that chronic conditions or medication use among older adults may also increase vulnerability [14], but we did not find a difference in the risk of heat stroke admissions during heat waves across the three age groups. One possible explanation could be that those individuals who are older or most susceptible to heat-related illness may be more likely to avoid activities (e.g. walking outside) that would increase their risk during very hot days.

Our estimate of the overall RR over the study period was 11.0 (8.8–13.6) on heat wave days compared to matched non-heat wave days. Bobb et al. (2014) reported a smaller RR of 2.54 (2.14–3.01) for the effect of heat wave (>99th, at least two days) on a disease category that included heat stroke, heat exhaustion, and other heat-related illnesses for the same study period [2]. The reason why the previous study yielded a much smaller estimate is because they used the broader disease grouping, which included not only heat stroke and sunstroke but also various other diagnoses that are not specifically related to heat, while we focused solely on heat stroke.

There are several limitations in our study. First, some of the counties in the west US are much larger than the ones in the eastern US. Assigning an average temperature to large counties using the temperature data from only a few monitoring sites is not optimal. In addition, using only a few monitoring sites to assess temperature in a county is likely to produce Berkson errors, which, although it does not create bias, could widen the confidence intervals [32]. Many counties relied on only a few monitoring sites, which also makes it difficult to weight temperature by population. Future satellite-based temperature prediction models, which could predict temperature up to 1 km spatial resolution, could address this issue [33]. Second, NDVI is also not a perfect measure of vegetation coverage or the cooling effects by the vegetation. Third, AC prevalence, an ecological measure, was used instead of actual AC use. Third, although the matching algorithm considerably reduced the computation time and removed the confounding by seasonality and county-level time-invariant covariates, the control days were randomly picked from a number of potential candidates and such a randomness could be passed into the results of the modeling.

## Conclusion

In conclusion, the risk of heat stroke has decreased substantially over time. However, considerable risk still remained through 2010. The substantial difference in the effect of heat-related disease risk across regions implies that public health policies for extreme heat events need to be regional. Among a number of covariates, AC has the potential to decrease the RR. An increase in AC prevalence in areas such as the northeast could be one of the possible strategies to further reduce the risk. Additionally, the RR was higher for longer and more intense heat waves. Such an excess risk is likely to be reduced by implementing an early warning system for heat waves in areas that lack one. This would be particularly helpful as heat waves are expected to be longer and more intense in the future.

## Abbreviations

AC, air conditioning; AHS, American Housing Survey; EPA, Environmental Protection Agency; HCC, high cloud cover; ICD-9, the International Classification of Diseases, Ninth Revision; IMPROVE, Interagency Monitoring of Protected Visual Environment; LCC, low cloud cover; MCC, medium cloud cover; Metro, Metropolitan areas; MODIS, Moderate Resolution Imaging Spectroadiometer; NASA, National Aeronautics and Space Administration; NCAR, National Center for Atmospheric Research; NCDC, National Climatic Data Center; NCEI, National Centers for Environmental Information; NCEP, National Center for Environmental Protection; NDVI, Normalized Difference Vegetation Index; NIH, National Institute of Health; Non-M 1, non-metropolitan with population >20,000; Non-M 2, non-metropolitan with population < 20,000; RH, relative humidity; RR, relative risk; T, temperature; WS, wind speed

## Additional file

Additional file 1: Supplementary Materials. **Text 1.** Detailed methods on covariates: temperature, RH, wind speed, dew point temperature NDVI, ozone concentration, cloud cover, air conditioning data. **Text 2.** Detailed methods on testing effect modifications. **Figure S1.** The RR of heat stroke on heat wave days compared to matched non-heat wave days in June, July, and August. The models controlled for indicator variables of year and day of the week. **Figure S2.** Temporal trends of log RR of heat stroke on heat wave days compared to matched non-heat wave days in (a) central, (b) east north central, (c) northeast, (d) northwest, (e) south, (f) southeast, (g) southwest, and (h) west. The time trends were estimated by natural splines with three degrees of freedom, controlling for indicator variables of day of the week. The model for the west north central did not converge because the number of cases was too few. The model for the southwest is not as stable as other regions due to the sparsity of the outcome. **Figure S3.** The RR of heat stroke on heat wave days compared to matched non-heat wave days in for Medicare beneficiaries in 65–74, 75–84, and >84 years in age. The models controlled for indicator variables of year and day of the week. **Figure S4.** Relative change in RR [exp (unit change*coefficient for the modifier)] of heat stroke on heat wave days compared to matched non-heat wave days per 10° Fahrenheit increase in daily temperature (T), per 10° Fahrenheit increase in daily dew point temperature (DEWP), per 10 % increase in relative humidity (RH), per 10 % increase in low cloud cover (LCC), per 10 % increase in medium cloud cover (MCC), per 10% increase in high cloud cover (HCC), and the temperature percentile before heat wave event (comparing 80–90^th^ and >90^th^ with <80^th^). The models controlled for the indicator variables of year and day of the week. **Figure S5.** Same as Fig. 3 panel (a) except that three other heat wave definitions were used (a) >98^th^ percentile temperature for at least two days, (b) >99^th^ percentile temperature for at least two days, (c) >97^th^ percentile for at least two days. **Figure S6.** Same as Fig. 3 panel (a) except that a quasi-Poisson model was fitted to allow overdispersion. (DOCX 637 kb)

## References

[1] Ye X, Wolff R, Yu W, Vaneckova P, Pan X, Tong S (2012). Ambient temperature and morbidity: a review of epidemiological evidence. Environ Health Perspect.

[2] Bobb JF, Obermeyer Z, Wang Y, Dominici F (2014). Cause-specific risk of hospital admission related to extreme heat in older adults. Jama.

[3] Semenza JC, McCullough JE, Flanders WD, McGeehin MA, Lumpkin JR (1999). Excess hospital admissions during the July 1995 heat wave in Chicago. Am J Prev Med.

[4] Huang C, Barnett AG, Wang X, Vaneckova P, FitzGerald G, Tong S (2011). Projecting future heat-related mortality under climate change scenarios: a systematic review. Environ Health Perspect.

[5] Matthies F, Menne B (2009). Prevention and management of health hazards related to heatwaves. Int J Circumpolar Health.

[6] Knowlton K, Rotkin-Ellman M, Geballe L, Max W, Solomon GM (2011). Six climate change-related events in the United States accounted for about $14 billion in lost lives and health costs. Health Aff (Project Hope).

[7] Seneviratne SI, Donat MG, Mueller B, Alexander LV (2014). No pause in the increase of hot temperature extremes. Nature Clim Change.

[8] Perkins SE, Alexander LV, Nairn JR (2012). Increasing frequency, intensity and duration of observed global heatwaves and warm spells. Geophys Res Lett.

[9] Field CB: Managing the risks of extreme events and disasters to advance climate change adaptation: special report of the intergovernmental panel on climate change: Cambridge University Press; 2012 10.1136/jech-2012-201045 22766781

[10] IPCC (2013). Climate Change 2013: The Physical Science Basis. Contribution of Working Group I to the Fifth Assessment Report of the Intergovernmental Panel on Climate Change.

[11] White-Newsome JL, Ekwurzel B, Baer-Schultz M, Ebi KL, O'Neill MS, Anderson GB (2014). Survey of county-level heat preparedness and response to the 2011 summer heat in 30 U.S. States. Environ Health Perspect.

[12] Argaud L, Ferry T, Le QH, Marfisi A, Ciorba D, Achache P, Ducluzeau R, Robert D (2007). Short- and long-term outcomes of heatstroke following the 2003 heat wave in Lyon, France. Arch Intern Med.

[13] Bouchama A, Knochel JP (2002). Heat stroke. N Engl J Med.

[14] CDC: Heat Stress in Older Adults. http://emergency.cdc.gov/disasters/extremeheat/older-adults-heat.asp. Accessed: Aug 6th, 2015. 2015.

[15] Knowlton K, Rotkin-Ellman M, King G, Margolis HG, Smith D, Solomon G, Trent R, English P (2009). The 2006 California heat wave: impacts on hospitalizations and emergency department visits. Environ Health Perspect.

[16] Ostro B, Rauch S, Green R, Malig B, Basu R (2010). The effects of temperature and use of air conditioning on hospitalizations. Am J Epidemiol.

[17] Naughton MP, Henderson A, Mirabelli MC, Kaiser R, Wilhelm JL, Kieszak SM, Rubin CH, McGeehin MA (2002). Heat-related mortality during a 1999 heat wave in Chicago. Am J Prev Med.

[18] Bobb JF, Peng RD, Bell ML, Dominici F (2014). Heat-related mortality and adaptation to heat in the United States. Environ Health Perspect.

[19] Karl T, Koss WJ: Regional and national monthly, seasonal, and annual temperature weighted by area, 1895–1983: National Climatic Data Center; 1984

[20] Ballester J, Robine JM, Herrmann FR, Rodo X (2011). Long-term projections and acclimatization scenarios of temperature-related mortality in Europe. Nat Commun.

[21] Nordio F, Zanobetti A, Colicino E, Kloog I, Schwartz J (2015). Changing patterns of the temperature-mortality association by time and location in the US, and implications for climate change. Environ Int.

[22] Lee M, Nordio F, Zanobetti A, Kinney P, Vautard R, Schwartz J (2014). Acclimatization across space and time in the effects of temperature on mortality: a time-series analysis. Environ Health.

[23] Baccini M, Biggeri A, Accetta G, Kosatsky T, Katsouyanni K, Analitis A, Anderson HR, Bisanti L, D'Ippoliti D, Danova J (2008). Heat effects on mortality in 15 European cities. Epidemiology (Cambridge, Mass).

[24] Greene JS, Kalkstein LS (1996). Quantitative analysis of summer air masses in the eastern United States and an application to human mortality. Clim Res.

[25] O’Neill MS, Zanobetti A, Schwartz J (2005). Disparities by race in heat-related mortality in four US cities: the role of air conditioning prevalence. J Urban Health.

[26] Shashua-Bar L, Hoffman ME (2000). Vegetation as a climatic component in the design of an urban street: An empirical model for predicting the cooling effect of urban green areas with trees. Energy Build.

[27] Peng S, Piao S, Ciais P, Friedlingstein P, Ottle C, Bréon F-M, Nan H, Zhou L, Myneni RB (2012). Surface urban heat island across 419 global Big cities. Environ Sci Technol.

[28] Anderson BG, Bell ML (2009). Weather-related mortality: how heat, cold, and heat waves affect mortality in the United States. Epidemiology (Cambridge, Mass).

[29] Ren C, Williams GM, Morawska L, Mengersen K, Tong S (2008). Ozone modifies associations between temperature and cardiovascular mortality: analysis of the NMMAPS data. Occup Environ Med.

[30] Anderson GB, Bell ML (2011). Heat waves in the United States: mortality risk during heat waves and effect modification by heat wave characteristics in 43 U.S. communities. Environ Health Perspect.

[31] Hajat S, Armstrong B, Baccini M, Biggeri A, Bisanti L, Russo A, Paldy A, Menne B, Kosatsky T (2006). Impact of high temperatures on mortality: is there an added heat wave effect?. Epidemiology (Cambridge, Mass).

[32] Zeger SL, Thomas D, Dominici F, Samet JM, Schwartz J, Dockery D, Cohen A (2000). Exposure measurement error in time-series studies of air pollution: concepts and consequences. Environ Health Perspect.

[33] Shi L, Liu P, Kloog I, Lee M, Kosheleva A, Schwartz J (2015). Estimating daily air temperature across the Southeastern United States using high-resolution satellite data: A statistical modeling study. Environ Res.

