# Supplementary material for: Heat stroke admissions during heat waves in 1,916 US counties for the period from 1999 to 2010 and their effect modifiers
Source: Environ Health. 2016 Aug 8;15:83. doi: 10.1186/s12940-016-0167-3 (PMC4977899; doi:10.1186/s12940-016-0167-3)
Supplement: Supplementary file 1 — Supplementary Materials. Text 1. Detailed methods on covariates: temperature, RH, wind speed, dew point temperature NDVI, ozone concentration, cloud cover, air conditioning data. Text 2. Detailed methods on testing effect modifications. Figure S1. The RR of heat stroke on heat wave days compared to matched non-heat wave days in June, July, and August. The models controlled for indicator variables of year and day of the week. Figure S2. Temporal trends of log RR of heat stroke on heat wave days compared to matched non-heat wave days in (a) central, (b) east north central, (c) northeast, (d) northwest, (e) south, (f) southeast, (g) southwest, and (h) west. The time trends were estimated by natural splines with three degrees of freedom, controlling for indicator variables of day of the week. The model for the west north central did not converge because the number of cases was too few. The model for the southwest is not as stable as other regions due to the sparsity of the outcome. Figure S3. The RR of heat stroke on heat wave days compared to matched non-heat wave days in for Medicare beneficiaries in 65–74, 75–84, and >84 years in age. The models controlled for indicator variables of year and day of the week. Figure S4. Relative change in RR [exp (unit change*coefficient for the modifier)] of heat stroke on heat wave days compared to matched non-heat wave days per 10° Fahrenheit increase in daily temperature (T), per 10° Fahrenheit increase in daily dew point temperature (DEWP), per 10 % increase in relative humidity (RH), per 10 % increase in low cloud cover (LCC), per 10 % increase in medium cloud cover (MCC), per 10% increase in high cloud cover (HCC), and the temperature percentile before heat wave event (comparing 80–90th and >90th with <80th). The models controlled for the indicator variables of year and day of the week. Figure S5. Same as Fig. 3 panel (a) except that three other heat wave definitions were used (a) >98th percentile temperature for at least two d [file 12940_2016_167_MOESM1_ESM.docx]

**Supplementary Material**

*for*

**Heat Stroke Admissions during Heat Waves in 1,916 US Counties for the period from 1999 to 2010 and Their Effect Modifiers**

*by*

Yan Wang, Jennifer F. Bobb, Bianca Papi, Yun Wang, Anna Kosheleva, Qian Di, Joel D. Schwartz, and Francesca Dominici

**Table of Contents Page**

**Text 1.**  Detailed methods on covariates: temperature, RH, wind speed, dew point temperature NDVI, ozone concentration, cloud cover, air conditioning data **3-4**

**Text 2.** Detailed methods on testing effect modifications **5-6**

**Figure S1.** The RR of heat stroke on heat wave days compared to matched non-heat wave days in June, July, and August. The models controlled for indicator variables of year and day of the week. **7**

**Figure S2.** Temporal trends of log RR of heat stroke on heat wave days compared to matched non-heat wave days in (a) central, (b) east north central, (c) northeast, (d) northwest, (e) south, (f) southeast, (g) southwest, and (h) west. The time trends were estimated by natural splines with three degrees of freedom, controlling for indicator variables of day of the week. The model for the west north central did not converge because the number of cases was too few. The model for the southwest is not as stable as other regions due to the sparsity of the outcome. **8**

**Figure S3.** The RR of heat stroke on heat wave days compared to matched non-heat wave days in for Medicare beneficiaries in 65 – 74, 75 – 84, and >84 years in age. The models controlled for indicator variables of year and day of the week. **9**

**Figure S4.** Relative change in RR [exp(unit change*coefficient for the modifier)] of heat stroke on heat wave days compared to matched non-heat wave days per 10° Fahrenheit increase in daily temperature (T), per 10° Fahrenheit increase in daily dew point temperature (DEWP), per 10% increase in relative humidity (RH), per 10% increase in low cloud cover (LCC), per 10% increase in medium cloud cover (MCC), per 10% increase in high cloud cover (HCC), and the temperature percentile before heat wave event (comparing 80 – 90^th^ and >90^th^ with <80^th^). The models controlled for the indicator variables of year and day of the week. **10**

**Figure S5.** Same as Figure 3 panel (a) except that three other heat wave definitions were used (a) >98^th^ percentile temperature for at least two days, (b) >99^th^ percentile temperature for at least two days, (c) >97^th^ percentile for at least two days. **11**

**Figure S6.** Same as Figure 3 panel (a) except that a quasi-Poisson model was fitted to allow overdispersion.  **12**

**Text 1.**

Methods that were applied to use temperature, RH, wind speed, dew point temperature, NDVI, ozone concentration, cloud cover, and air conditioning data are given below.

**Temperature, RH, wind speed, dew point temperature:** If a county on a day had at least one monitor running within that county, the average of the measurements from monitoring sites within that county was used. If a county on a day had no monitor running within that county but had at least one monitor within 35 km from its centroid, the average of the measurements from these monitors was used. Otherwise, a county was excluded from the analysis. The dataset before matching had 7,610,572 county-days in total. 62% of the county-day temperature was measured by one monitor, 25% was measured by two monitors, and 13% was measured by three or more monitors. We used both daily values of temperature, RH, wind speed, and dew point temperature, and the average of mean summer temperature, mean summer RH, and mean summer wind speed as a measure of a county’s climate to examine their effects on spatial contrast of the RR.

**NDVI:** A county’s NDVI was obtained by averaging all 1 km × 1 km grid cells whose centroids were within that county. The average of mean summer NDVI was used as a measure of the vegetation coverage in summer for that county to examine spatial contrast of the RR.

**Ozone concentration:** If a county on a day had at least one monitor running within that county, the average of the measurements from monitoring sites within that county was assigned. If a county on a day had no monitor running within that county, ozone concentration was regarded as missing. For ozone, we did not use the measurements from monitors within 35 km because of its large spatial variability. The average of mean summer ozone was used as a measure of average ozone pollution level for that county to examine its effect on spatial contrast of the RR. 716 out of 1916 counties had ozone monitors.

**Cloud cover (low/medium/high cloud cover):** Because of the low spatial resolution of cloud cover (~0.3 degree), we assigned each county to the cloud cover in the nearest grid cell from the reanalysis data set. We used both daily values of cloud cover and the average of mean summer cloud cover also as a measure of a county’s climate to examine its effect on spatial contrast of the RR.

**Air conditioning prevalence (central or any):[1]** If a county was surveyed, the prevalence of that county was assigned. Otherwise, AC prevalence was regarded as missing. 188 out of 1916 counties had AC data. A linear model was fitted to each county’s AC prevalence data. The air conditioning prevalence was interpolated and extrapolated over time in 1999-2010 using this linear model for the counties that were surveyed, and the average of AC prevalence was used to examine its effect on spatial contrast of the RR.

**Text 2.**

To explore which factors might modify the RR of heat stroke hospitalizations, we first investigated whether age group, as a measure of individual susceptibility, modified the RR. This is achieved by stratifying the daily hospitalizations (numerators) and Medicare beneficiaries (denominators) into three age groups (65 – 74, 75 – 84, >84 years) and including interaction terms of the heat wave day indicator and the age group indicator variables.

Second, for county-level covariates, we tested whether these variables explain the spatial contrast of the RR. Specifically, we fitted the following model,

log E(Y_ct_) = (γ_0,c_ + β_0_) + (β_1,c_ + γ_1,c_) HW_ct_ + β_2_ Dow_t_ + β_3_ Year_t_ + β_0,V_ V_c_ + β_1,V_ V_c_ HW_ct_ + log(P_ct_) [supp eq 1]

where V_c_ stands for one of the following county level covariates: the average of county-level AC prevalence (central or any) over the study period, mean summer NDVI, mean summer ozone concentration, mean summer temperature, mean summer RH, mean summer wind speed, mean summer low/medium/high cloud cover, or urbanicity of each county; and (γ_0,c_, γ_1,c_) follows a bivariate normal distribution with mean zero.

Third, we examined if the intensity and duration of heat wave alters the RR of heat stroke hospital admissions on heat wave days as compared to matched control days. This was achieved by comparing the effect of heat waves using the primary definition with using stricter definitions. More specifically, we compared the RR using six definitions of heat waves (>97^th^ for at least two days, >98^th^ for at least two days, >99^th^ for two days, >97^th^ for at least four days, >98^th^ for at least four days, >99^th^ for four days).

Fourth, we tested the interaction of heat wave with daily temperature, daily dew point temperature, daily RH, daily low, medium, high cloud cover, and the interaction between heat wave and the percentile of temperature on the day before heat wave to see whether a sharper increase in temperature was associated with a larger effect. Specifically, we used the following model,

log E(Y_ct_) = (γ_0,c_ + β_0_) + β_1,c_ HW_ct_ + β_2_ Dow_t_ + β_3_ Year_t_ + β_0,V_ V_ct_ + β_1,V_ V_ct_ HW_ct_ + log(P_ct_) [supp eq 2]

where V_ct_ is the daily meteorological variable.


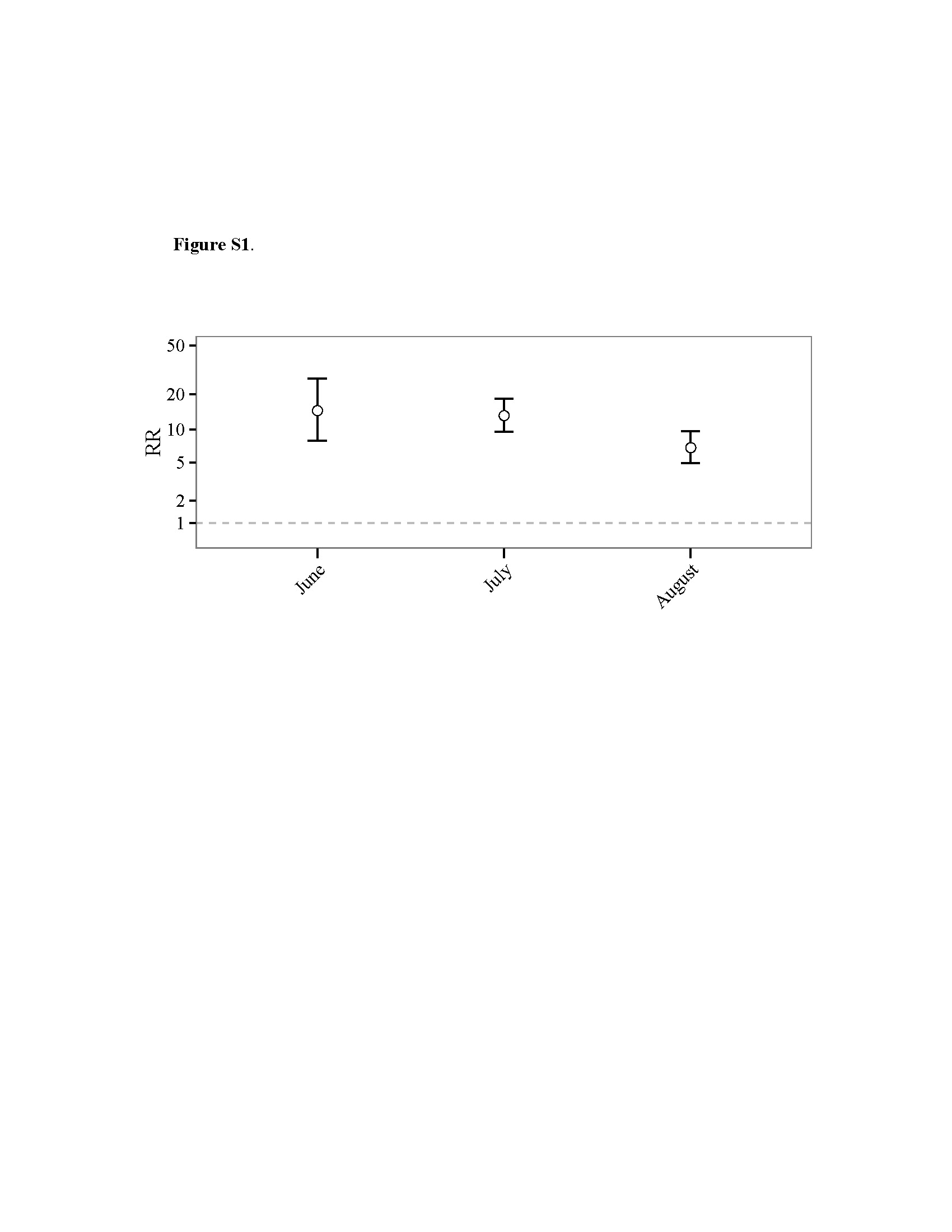


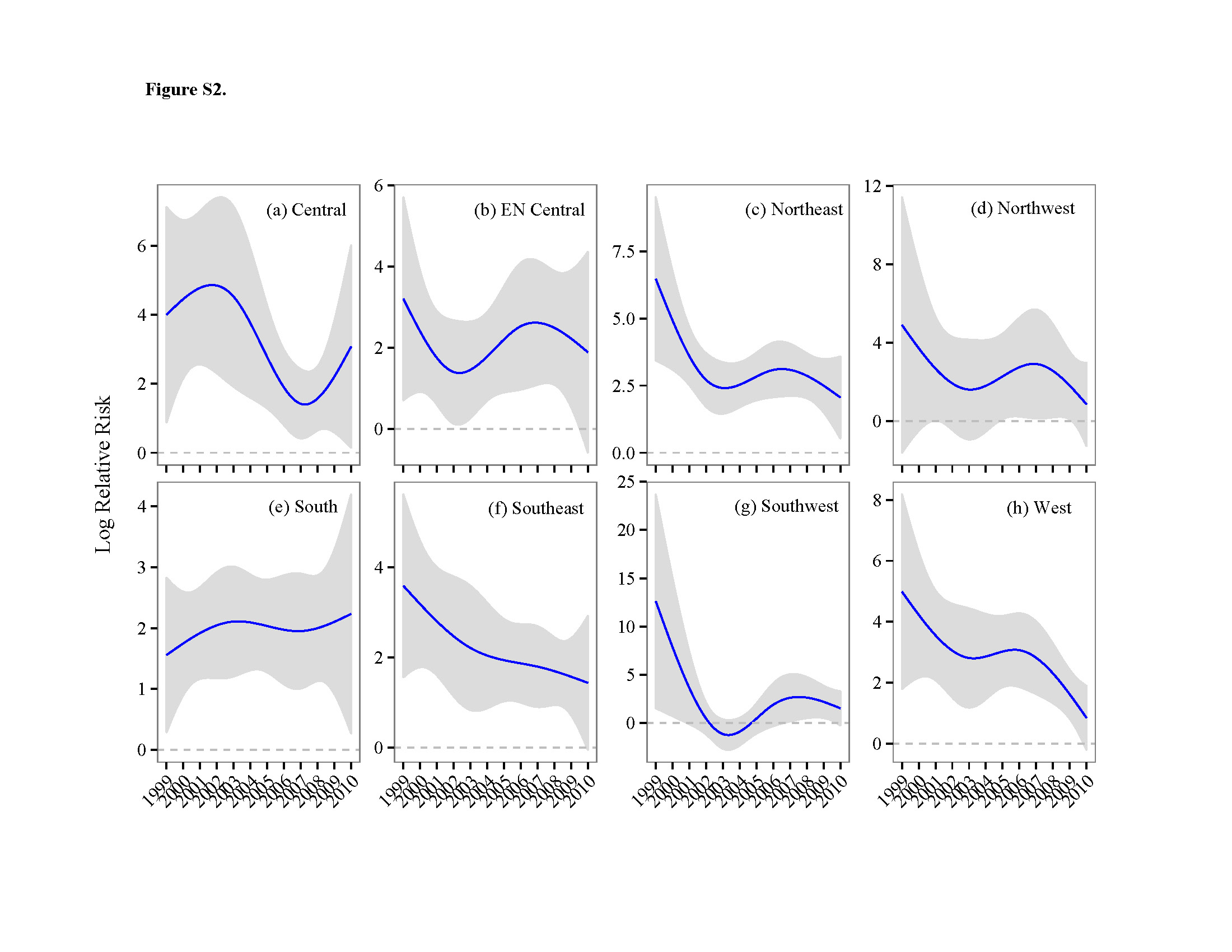


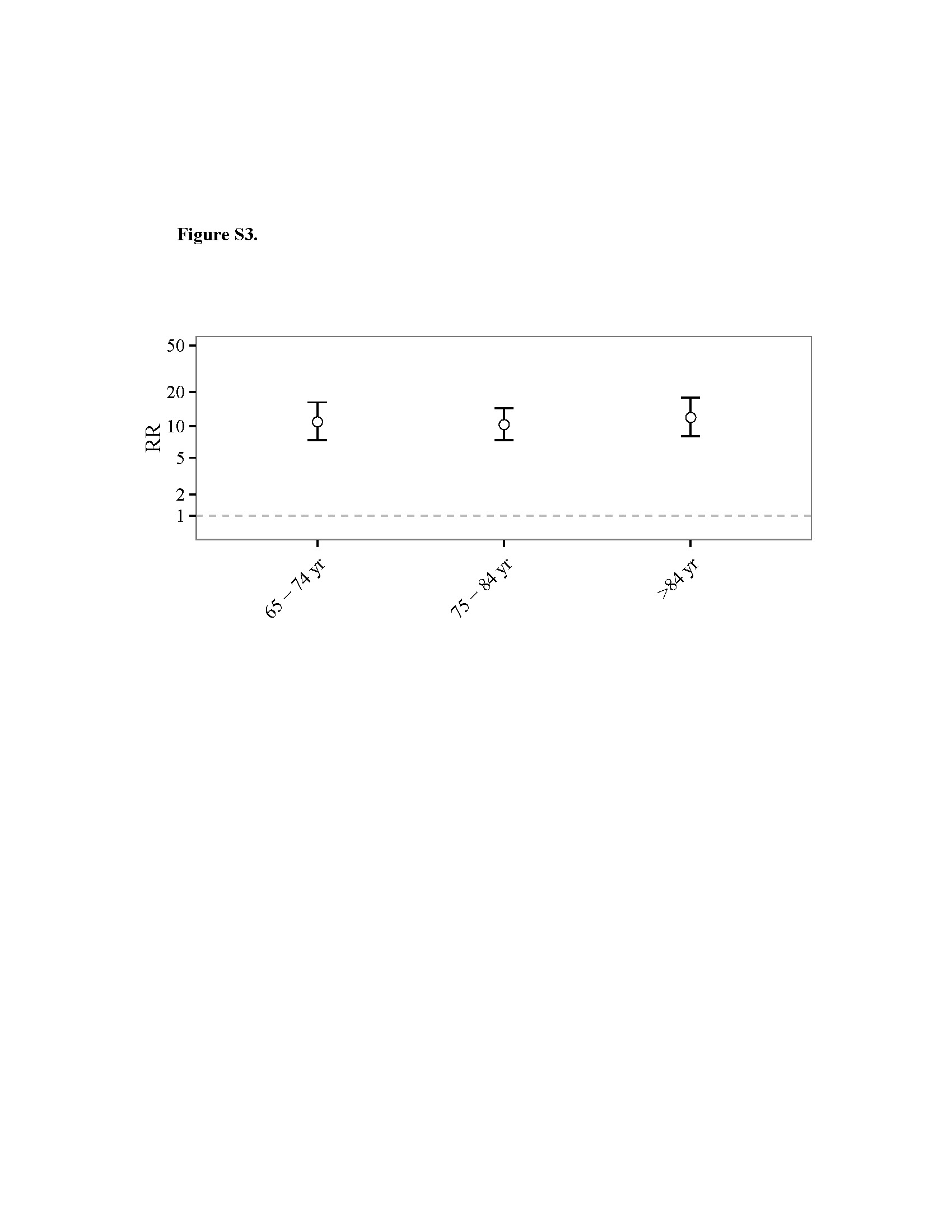


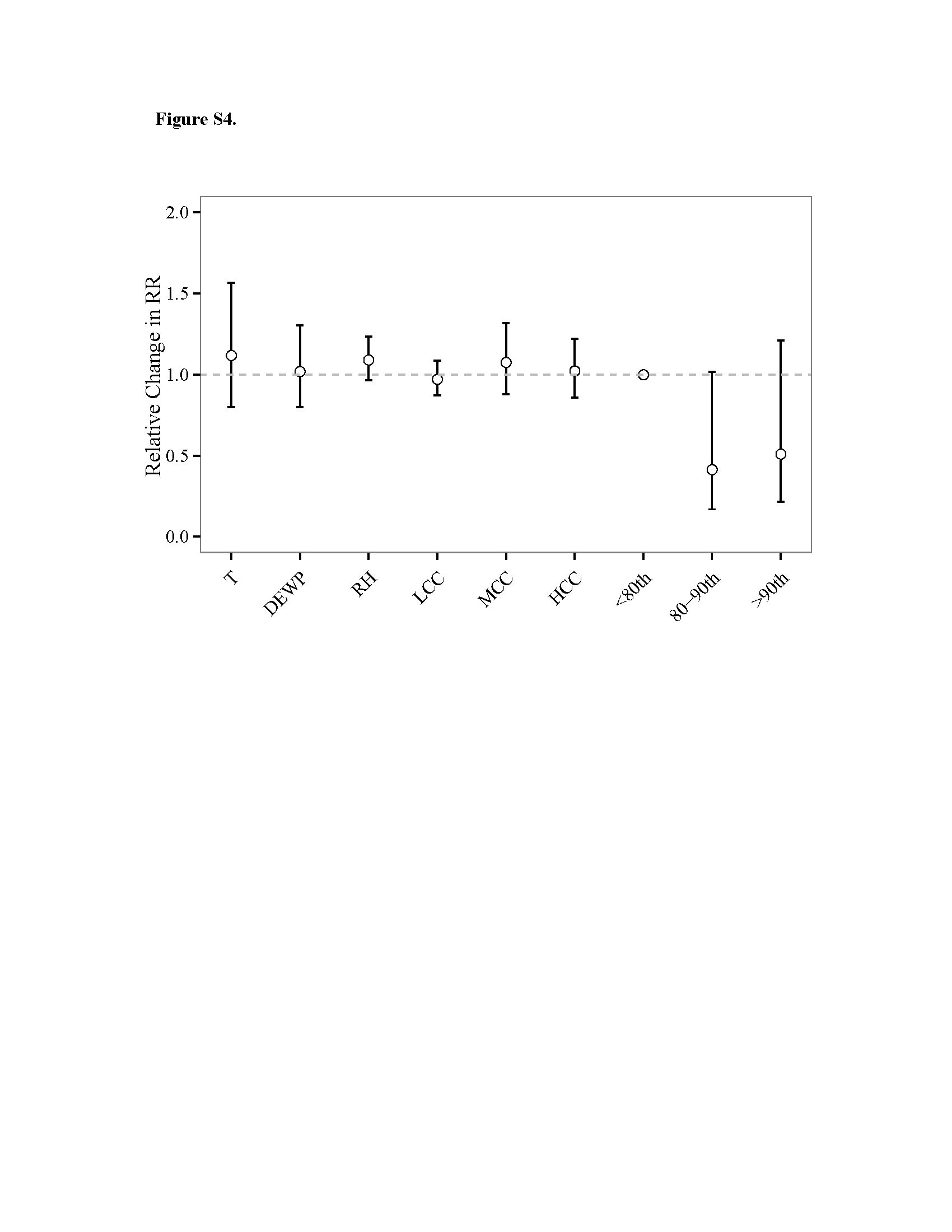


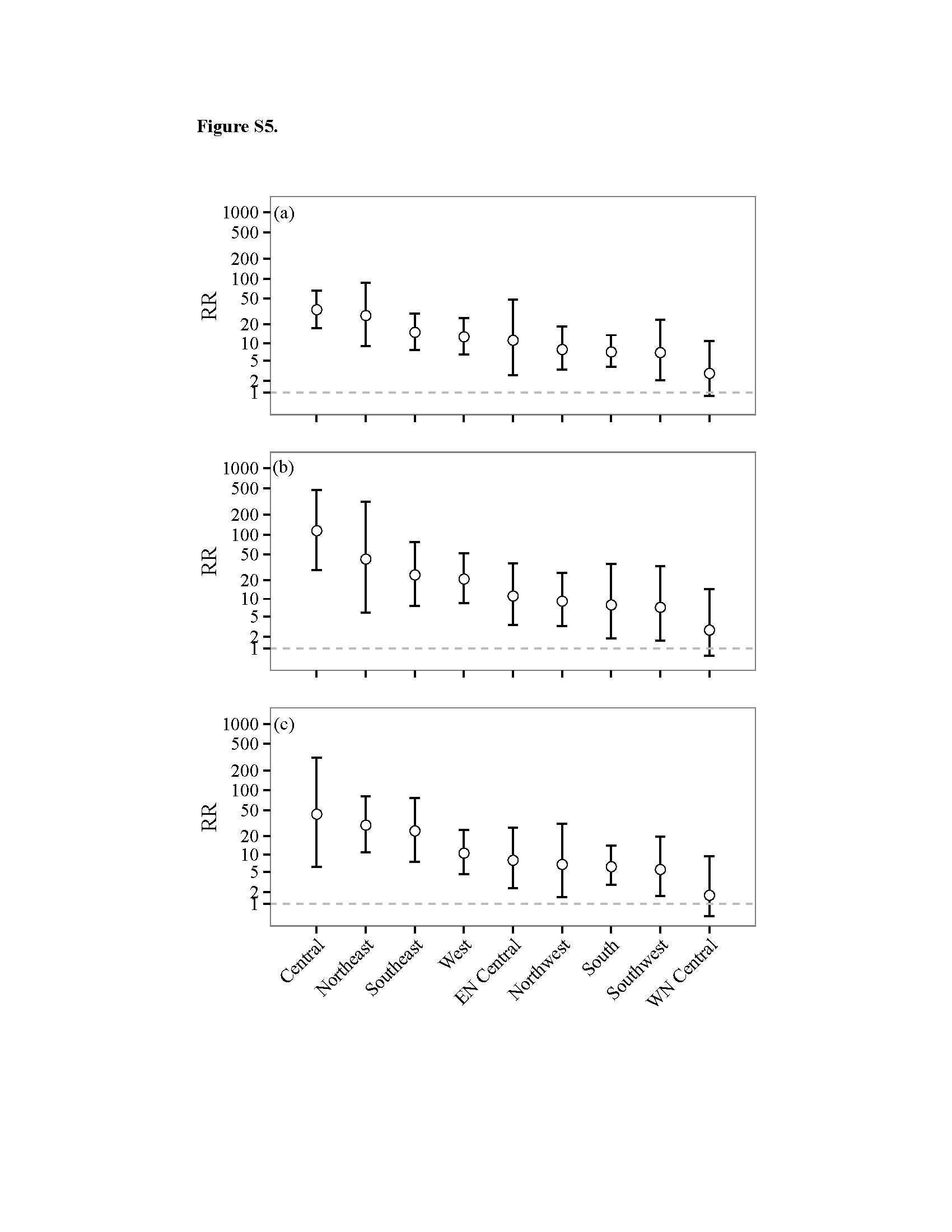


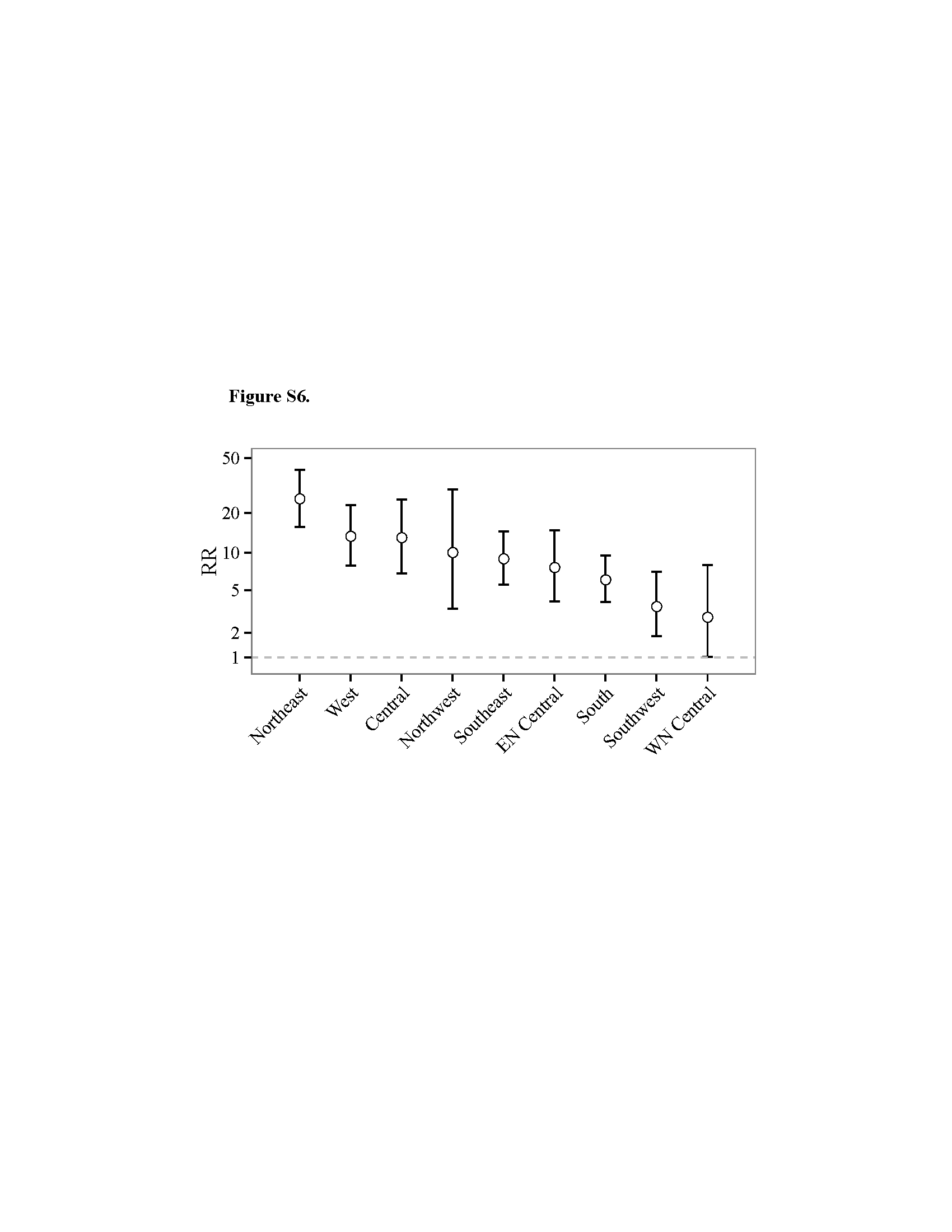


**References**

1. US Census Bureau: **American Housing Survey**. 2015. http://www.census.gov/programs-surveys/ahs/data.html
